# Food Addiction, Binge Eating Disorder, and Obesity: Is There a Relationship?

**DOI:** 10.3390/bs7030054

**Published:** 2017-08-14

**Authors:** Tracy Burrows, Janelle Skinner, Rebecca McKenna, Megan Rollo

**Affiliations:** 1 School of Health Sciences, Faculty of Health and Medicine, University of Newcastle, Callaghan 2308, Australia; Janelle.skinner@uon.edu.au (J.S.); Rebecca.Mckenna@uon.edu.au (R.M.); megan.rollo@newcastle.edu.au (M.R.); 2 Priority Research Centre for Physical Activity and Nutrition, University of Newcastle, Callaghan 2308, Australia

**Keywords:** binge eating, food addiction

## Abstract

Existing research suggests that there is an overlap between binge eating disorder (BED) and the construct of ‘food addiction’ (FA). The objective of this study was to determine the overlapping features of BED and FA through a comparison of the individual scales of commonly used tools including the Binge Eating Scale (BES) and the Yale Food Addiction Scale (YFAS) in a sample of Australian adults. Adults (>18 years of age) were invited to complete an anonymous online survey on FA. Binge eating was assessed through the BES and addictive eating behaviours were assessed through the YFAS (*n* = 1344). The prevalence and severity of both FA and binge eating increased across weight categories. The overall correlation between the total score from the BES and FA symptoms was *r* = 0.76, *p* < 0.001; for females it was *r* = 0.77, *p* < 0.001, and for males it was *r* = 0.65, *p* < 0.001. Total BES score and the BES emotion factor were most often associated with FA symptoms, as was demonstrated to produce stronger correlations with FA symptoms. In contrast, the BES behaviour factor was less strongly associated to FA with the majority of correlations <0.6. This study demonstrates the overlap between BED and FA, and highlights the possible unique differences between the forms of disordered eating.

## 1. Introduction

‘Food addiction’ (FA) presents as a contentious construct, which has yet to gain scientific acceptance due to an overall lack of high quality research [1]. There is scientific debate about the appropriateness of terminology, with ‘eating addiction’ previously proposed to be more reflective rather than ‘food addiction’, given the uncertainty of whether addictive eating behaviours more closely align to substance-based addictions, like drug and alcohol addictions, or with behavioural addictions, such as gambling addiction [2]. Addictions to drugs, alcohol, and gambling are formally recognised in the Diagnostic Statistics Manual, version 5 (DSM-5) [2]. However, FA is currently not a recognised condition in DSM and instead is characterised through the assessment and endorsement of addiction-like symptoms through self-report tools or surveys. The most commonly used tool is the Yale Food Addiction Scale (YFAS). Originally developed in 2009 and revised in 2016, the YFAS 2.0 maps to the criteria used to classify substance dependence [3]. The YFAS tool assesses 11 symptoms of addiction, as well the level of distress associated with them, which parallel other addiction symptoms in DSM such as tolerance and craving. In addition, the tool can determine the level of severity of addiction ranging from mild to severe [4].

There has been previous suggestion that FA may be a sub-type of disordered eating, and that the FA construct is an indicator of higher eating disorder severity [5]. Research suggests that FA does show overlap with several disordered eating phenotypes, with the majority of existing research investigating binge eating disorder (BED) and bulimia nervosa rather than other disordered eating categories such as anorexia nervosa [6,7].

BED is classified by the DSM-5 as the recurrent, periodic, and uncontrolled consumption of large quantities of food without compensatory behaviours (e.g., purging, laxative use) to control weight [8]. It has been estimated that BED affects approximately 2% of the global population [9], while FA affects approximately 20%, with both conditions found to be more common in females [1,10]. The prevalence of FA in two existing studies of individuals with diagnosed BED was 41.5% and 56.8% [11,12]. The prevalence of FA in individuals with a current diagnosis of bulimia nervosa was 83.6% and 100%, and 30% of individuals with a history of bulimia nervosa met the diagnostic criteria for FA [13,14]. In a more recent study of individuals with clinical BED assessed through an objective clinical interview rather than self-report, those with BED were also identified with FA (33.8%). Existing research suggests an overlap between these conditions, often through cross-sectional studies, which show that the overlap between BED and FA varies between 0.59 and 0.78) [1]. The overlap between BED and FA however is not 100%, and while binge eating is a key eating disorder feature, the association between FA and disordered eating behaviour is unclear. It is acknowledged that the symptoms of general psychological distress found to be associated with FA are strongly associated with binge eating and other eating disorder symptoms [15,16].

Similarities in symptomology that exist between FA and BED include the consumption of larger amounts of food than intended, reduced control over eating and continued use despite negative consequences [13,17], intense cravings [18], emotional dysregulation, and increased impulsivity [5,6,19]. Due to these similarities, it is not unusual that BED and FA are often highly correlated. A meta-analysis also reported that YFAS symptom scores were positively associated with binge eating behaviours [1].

Existing research has investigated FA specifically in binge eating populations and individuals seeking bariatric surgery, and identified similarities in the specific characteristics of both conditions when assessed by validated surveys [20,21]. In addition, recent research highlights the associations between patterns of compulsive overeating, including binge eating with ‘food addiction’ [22]. However, these conditions have not been investigated at the scale, factor, or item level of common assessment scales. Instead previous research has explored associations in absolute scores that indicate the overall severity of FA or BED. Investigating factor levels for these constructs may provide important information about where the overlapping features exist and whether there are factors which separate these conditions. Strong correlations would be expected between subscales that may map to the same attribute, and lower correlations between subscales from different attributes. Therefore, the current study aims to determine the overlapping features of BED and FA through a comparison of the individual constructs of the Binge Eating Scale (BES) and YFAS in a sample of Australian adults. 

## 2. Materials and Methods

Participants aged 18 years or above and living in Australia were invited to complete an anonymous online survey on FA. The survey took approximately 20 min to complete. Exclusion criteria included being pregnant/currently lactating and being unable to comprehend English. Recruitment was undertaken over a three month period in 2016. The study was advertised through University of Newcastle media releases and promoted via a variety of social media platforms (i.e., Facebook, Twitter). The advertisements contained a link to the survey, and participants provided informed consent before completing the survey. Survey completers were invited to enter a prize draw to win 1 of 10 shopping vouchers ($50 value). This study was approved by The University of Newcastle Ethics Committee.

The survey comprehensively assessed a range of measures relating to diet and mental health which has been previously reported [15]. The current study is a secondary analysis relating specifically to FA and BED status. Demographic information was assessed through 10 items and included information on gender, age, ethnicity, marital status, postcode, highest level of education, height, and weight, which was converted into body mass index (BMI) using standardised equations. Participants’ BMI was then classified according to World Health Organisation classifications [23]. Postcode was used to determine the Index of Relative Socioeconomic Advantage and Disadvantage (ISRAD), where postcode is rated from one (most disadvantage/least advantage) to 10 (least disadvantaged/most advantaged).

FA was assessed using the 35-item YFAS 2.0 [4]. Each question offers a participant an option of eight responses ranging from ‘never’ to ‘every day’. Each symptom is considered met when one or more of the relevant questions for each criteria meet a predefined threshold. It provides a ‘diagnosis’ of FA which, depending on the number of symptoms endorsed, can be classified as ‘mild’, ‘moderate’ or ‘severe’. A mild diagnosis score is given when 2–3 symptoms are reported, moderate when 4–5 symptoms are present, and severe when 6 or more symptoms. Symptoms include tolerance, withdrawal, and loss of control with respect to eating behaviour. The YFAS 2.0 asks participants to think of specific foods such as highly processed foods; however, participants in this study were asked to consider all food. In the current study, the Cronbach alpha for this tool was 0.95, indicating acceptable internal consistency.

Binge eating was assessed through the standardised BES, which comprises 16 questions. Each question requires a response consisting of three to four possible responses, reflecting a range of severity. The total score is tallied to give a score out of 46, with higher scores representing increased binge status. Based on the total score, individuals can be classified as ‘no binge eating’ if the score is ≤17, ‘mild to moderate binge eating’ if the score is 18–26, and ‘severe binge eating’ if the score is >27. The BES has been previously shown to have a two-factor structure, which was originally demonstrated by Gormally et al. [24] and confirmed more recently by Kelly et al. [25]. These factors relate to (1) behavioural manifestations (eight items) including factors such as eating large amounts of food, and (2) feelings and cognitions (eight items) surrounding a binge eating episode including guilt, fear of not being able to stop eating, and preoccupation with eating. For the current study, in addition to the total score, the two-factor scores were also determined according to author instructions. The BES is not designed as a direct measure of BED [26]. In the current, study the Cronbach alpha for this tool was 0.92, indicating acceptable internal consistency. 

*Statistics*: Descriptive statistics were undertaken, *t*-tests were used to examine differences between groups (food addicted vs. non-food addicted or females vs. males). ANOVA were used to determine differences in addiction severity (mild, moderate, severe). Correlation matrices for the scales for both YFAS and BES were undertaken. Also, Spearman and Pearson correlations were determined in the cases where data distribution was not normal. Both correlations produced similar results; associations between factors of the BES and between symptoms of the YFAS are presented using Pearson correlations. Correlations were determined as small (0.1–0.2), medium (0.3–0.5), or large (>0.6). The correlation analysis between scales used for this study has been undertaken in previous health research [27]. The results presented are for complete cases, with 869 of the 1344 respondents who started the survey having answered all questions. A missing value analysis was undertaken, and the demographics of those with missing values were compared, with no patterns identified. Therefore, there was no imputation of data for missing values. Due to the multiple statistical tests completed as part of this analysis, data were adjusted using a Bonferroni correction with a lower statistical threshold, and a statistical significance of *p* < 0.01. Data was analysed using SPSS version 22.0 (SPSS Inc., Chicago, IL, USA). 

## 3. Results

### 3.1. Participants

The survey recruited *n* = 1344 individual; 80.7% were female and 19.3% were male. The demographic details can be found in Table 1. The mean ISRAD score was 6.2 ± 2.9, reflecting a moderate socioeconomic status; however, this value varied from 1–10, reflecting a moderately diverse population group. A total of 44.3% of the sample were married, 27% were never married, and 14.5% had been married but were without a current partner. The population comprised 1.6% with a trade/apprenticeship, 20% with a certificate/diploma, 36% with a university degree, and 31% with a higher education including masters, Ph.D. 

### 3.2. Food Addiction and Binge Eating 

Across the whole sample, the prevalence of FA was found to be 22.2% (*n* = 228). This differed significantly by gender, with females (24.4%) having a higher prevalence than males (13.3%; *p* < 0.001). According to the YFAS 2.0 categorisations of severity, the majority of individuals classified as severely food addicted 18.9% (*n* = 194), while 2.6% (*n* = 27) were moderately addicted, and <1% were classified as mild. The prevalence and severity of both FA and binge eating increased across weight categories (Table 2). 

For those who had completed the BES, the mean BES score was 11.9 ± 9.3 (range 0–42). For males the mean BES scores was 9.2 ± 7.4 (range 0–36), and for females the mean score was 12.6 ± 9.6 (range 0–42). The mean values for each sex were significantly different (*p* < 0.01). For the BES, severity for the total group was determined, with 74% of individuals classified as non-binge eaters (*n* = 660), 17.4% (*n* = 155) moderate binge eaters, and 8.7% (*n* = 78) severe binge eaters. Differences in BES severity were determined by sex, and significant differences were found between males and females. Among non-binge eaters, males accounted for 86.2% vs. females 70.9%, among moderate binge eaters it was males 12.1% vs. females 18.6%, and among severe binge eaters it was males 1.7% vs. females 10.4%, all *p* < 0.01. Individuals with FA reported significantly higher on the BES total, emotions, and behavioural scales than those who were non-food addicted (Table 3). Of those with FA, 72.7% reported scores of either moderate (41%) or severe (31.7%) bingeing compared with only 9.9% in the non-food addicted group. A significant correlation was found between the two-factor scores of behaviours and emotions of the BES: *r* = 0.82, *p* < 0.001 (Table 4). 

### 3.3. Relationships between Food Addiction and Binge Eating 

**Figure 1:**
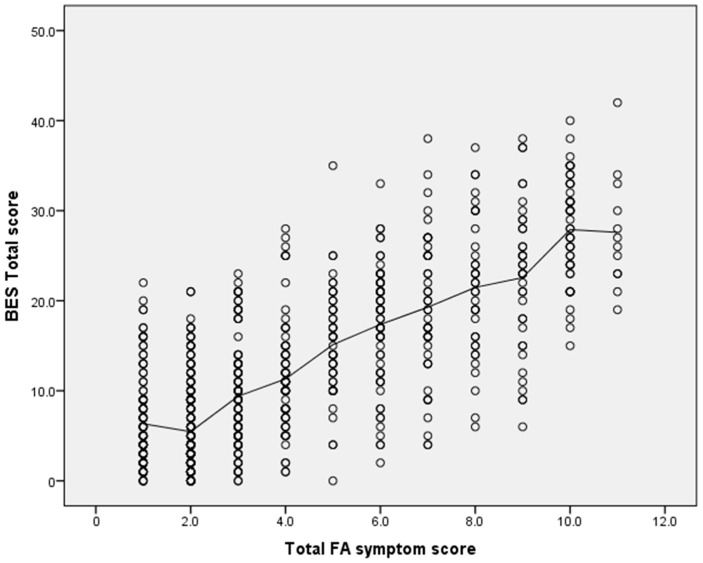
Correlation between BES total scores and YFAS symptom scores.

The overall correlation between the total score from the BES and FA symptoms was *r* = 0.76, *p* < 0.001; for females it was *r* = 0.77, *p* < 0.001, and for males it was *r* = 0.65, *p* < 0.001 (Figure 1). Total BES score and BES emotion factor were more strongly associated with FA symptoms, as evidenced by the majority of correlations (*n* = 7) with values >0.6. In contrast, the BES behaviour factor was less strongly associated to FA, as evidenced by smaller correlation values, with only three correlations classified as large (*r* > 0.6, *p* < 0.001). Of the 11 individual FA symptoms and clinical impairments, correlations with BES factors and the FA symptoms ‘consumed more than planned’ were small, with the majority being <0.3, while ‘use in physically hazardous situations’ produced significant negative correlations. 

## 4. Discussion

This study investigated the overlap in symptoms of FA and BED as measured by the YFAS 2.0 and the BES’s emotional and behavioural factors. It was found that substantial overlap (*r* = 0.76) exists between the commonly used assessment tools in a sample of Australian adults from a wide age range, which concurs with existing research [28,29]. However, this is the first study to demonstrate that the strongest overlap occurred with the BES emotion factor, while less overlap was observed with the BES behaviour factor. This was evidenced by larger correlation values with the BES emotion factor, compared with very few strong correlations (greater than 0.6) with the BES behaviour factor. The behaviour factor relates to behavioural expressions surrounding a binge eating episode which showed small to moderate correlations with the majority of the 11 symptoms assessed by the YFAS 2.0 tool. These correlations are not unexpected, as previous findings indicate that individuals with co-existing FA and BED experience significantly higher levels of depression, negative affect, poorer emotion dysregulation, and lower self-esteem [11]. This would seem to indicate that high levels of emotional and psychosocial distress accompany both eating pathologies. Research has demonstrated that, in the context of binge eating, emotions and behaviour rarely occur exclusively [30] and are correlated as shown in this study. For this reason, it would be expected that both features be present and it is unsurprising that these symptoms would co-occur in FA. However, strong evidence for the cognitive and behavioural aspects of BED is lacking, and further investigations into BED and behaviour are warranted.

It is important to attempt to articulate the unique features that may set the construct of FA apart from BED, given that previous overlaps have been shown, although these are not in entirety or not 100% overlapping. In this study, it appears that several symptoms are unique to FA, as evidenced by lower correlation valueswith BES. Specifically, small associations were found with FA symptoms of ‘consumed more than planned’ or ‘use in physically hazardous situations’; the latter symptom in this study actually showed significant negative relationships. The symptom of ‘use in physically hazardous situations’ has previously been debated as a difficult factor to interpret in relation to food, given that food in its true sense is needed for survival. Recent studies assessing FA using the YFAS 2.0 tool have found the endorsement of ‘use in physically hazardous situations’ symptom in three non-clinical samples (*n* = 1900) to have low endorsement rates ranging from 9.1% to 24.8%; with two of the studies reporting the endorsement rate in FA individuals (*n* = 109) as 37.0% and 68.3%, respectively [4,28,31]. However, in the context of FA, this symptom can be described as causing impairment to performance, such as eating while driving, or impairment to health that is hazardous. In the context of obesity and related metabolic syndrome risk factors, this could include the consequences of individuals with diabetes, dyslipidaemia, or hypertension overconsuming foods containing excessive amounts of sugar, fat, or sodium [32]. However, this symptom may not have been well-reported by participants with this rationale in mind nor understood in terms of addiction by the participants completing the surveys, thus influencing the results because the questions did not ask participants to consider this aspect of the symptom. Existing public views suggest that individuals believe some foods are addictive and that addiction can cause obesity [33]. Future qualitative work on FA is warranted to better understand how the symptoms are experienced and if they differ for each individual, particularly as this field is still emerging. 

The FA symptom of “loss of control with respect to eating behaviour” overlaps with that of “loss of control over eating”, the latter being a core eating disorder behaviour and a diagnostic criterion for the eating disorders bulimia nervosa and binge eating disorder [32]. The symptoms of FA as assessed by the YFAS were determined with mapping to the DSM–5. However, it is noted that when considering some of these symptoms at a broader population level, with increasing prevalence rates of overweight and obesity, some symptoms or traits presently being assessed overlap with general dieting practices undertaken by many individuals. FA symptoms measured by the YFAS, specifically ‘repeated attempts to cut down food’, may not be unique to FA, but apply to the population in general. 

In the current analysis of those individuals with FA, 72.7% also had reported BES scores which related to either moderate or severe bingeing. While not directly comparable due to the use of different tools and methods to assess the disordered eating status, the value in the current study is higher than that previously reported by Gearhardt et al., who found in a sample of overweight individuals with BED, determined by clinical interview, that 57% met the classification for FA [11]. In a more racially diverse sample of obese, treatment-seeking adults with BED (*n* = 96) as assessed by an alternate tool, The Eating Disorder Examination Questionnaire (EDEQ), the findings were similar, with 42% of participants meeting the classification for FA [12]. In both studies, YFAS scores were also significant predictors of binge eating frequency. In an additional study, Ivezaj et al. [34] examined the eating and health-related behaviours of overweight/obese adults (*n* = 502), and found that 61.7% of adults meeting BED criteria as assessed by the EDEQ also met FA criteria. Adults with co-occurring BED and FA had significantly higher BMI and depression scores, combined with greater disturbances on most impulsivity and self-control measures relative to the control group [35]. A strong association between FA and BE severity (*r* = 0.78, *p* = 0.0045) as well as a moderate association between FA and measures of general psychopathology were reported. A similar relationship between FA and BE has also been shown to exist in younger adolescent populations [35]. 

Rates of FA among those with BED is higher in individuals with obesity than in those who are not obese [36]; this was also shown in the current study across increasing weight status of healthy, overweight, and obese participants. Individuals who meet the criteria for both BED and FA tend to exhibit more frequent binge eating episodes, experience stronger cravings for food, and elevated levels of impulsivity and depressive symptoms than those with only BED [5,12,13]. It has been suggested the co-occurrence of BED and FA may represent a more severe BED subgroup characterised by greater eating disorder psychopathology and associated pathology [11].

Recent evidence suggests altered reward sensitivity may contribute to the pathophysiology of disordered eating behaviours. A review of neuroimaging studies (*n* = 15) in BED found that the alterations in corticostriatal circuitry were similar to those observed in substance abuse, including altered function of prefrontal, insular, and orbitofrontal cortices and the striatum [37]. Preliminary evidence by Gearhardt et al. suggests that reward dysfunction may also be a relevant mechanism in the FA construct [38]. Human genetics and animal studies suggest that changes in neurotransmitter networks, including dopaminergic and opioidergic systems, are associated with compulsive-eating behaviours [37,39].

This study has several limitations which should be considered when interpreting the findings: the survey was collected online and is based on self-report measures and was analysed using correlation analysis only. However, it is noted that for food addiction and binge eating, the majority of measures used for these eating behaviours are based on self-report, and self-reported height and weight have been shown previously to be a valid measure of weight status [40]. It could be likely that individuals who are motivated by food may have been more likely to complete the survey. The study sample had a majority of participants who were female, so results may not be generalisable to the broader population or to other ethnicities. The findings of this study have some clinical implications, as they provide a further understanding of the underlying aetiology of co-occurring FA and BED, to progress and tailor treatment options as FA appears to be more physiological than behavioural in nature. 

## 5. Conclusions

This is one of the first studies to investigate the potential overlap between the common tools used to assess BED and FA and their individual constructs, particularly with reference to the YFAS 2.0 tool which maps to current DSM criteria. This study demonstrates the overlap between BED and FA, and highlights the possible unique differences between the forms of disordered eating. 

## Figures and Tables

**Table 1:** Demographic details of samples according to food addiction status.

Demographic	NFA Male (*n* = 176 )	FA Male (*n* = 27 )	*p* Value	NFA Female (*n* = 624 )	FA Female (*n* = 201 )	*p* Value
	Mean ± SD	Mean ± SD		Mean ± SD	Mean ± SD	
**Age** (years)	42.0 ± 13.2	46.0 ± 16.4	0.531	39.6 ± 13.1	40.1 ± 13.0	0.407
**Height** (cm)	178.8 ± 0.1	179.7 ± 0.1	0.629	166.0 ± 0.1	165.9 ± 0.1	0.935
**Weight** (kg)	87.4 ± 15.4	113.7 ± 28.3	<0.01	70.0 ± 16.7	88.4 ± 22.9	<0.01
BMI * (kg/m^2^)	27.4 ± 4.8	35.4 ± 8.8	<0.01	25.5 ± 6.0	32.7 ± 13.1	<0.01
	*n* = 149	*n* = 22		*n* = 517	*n* = 183	
**BES total**	7.4 ± 5.8	20.1 ± 6.1	<0.01	8.5 ± 6.5	22.8 ± 8.1	<0.01

Note: Statistically significant differences between sex determined by *t*-tests. NFA, non-food addicted; FA, food addicted; BES, Binge Eating Survey. BMI body mass index

**Table 2:** Comparison of food addiction (FA) and Binge Eating (BE) according to BMI category ^a^.

Condition	Underweight (*n* = 14 )	Healthy (*n* = 439 )	Overweight (*n* = 263 )	Obese (*n* = 286 )	*p* Value
**Food Addiction (FA)**	%	%	%	%	
NFA	85.7	92.5	78.7	53.8	<0.001
Mild FA	0.0	0.9	0.4	0.7	0.368
Moderate FA	7.1	1.6	3.4	3.5	0.07
Severe FA	7.1	5.0	17.5	42.0	<0.001
**Binge Eating (BE)**					
Non-bingeing	90.0	89.3	74.8	50.6	<0.001
Moderate	10.0	7.5	19.5	30.4	<0.001
Severe	0.0	3.2	5.8	19.0	<0.001

Note: Data are shown as percentages within each group. Statistical significance was determined using ANOVA between weight categories ^a^ BMI categories (kg/m^2^): Underweight ≤18.50, Healthy = 18.50–24.99, Overweight = 25.00–29.99, Obese ≥30.00. FA, food addiction; NFA, non-food addicted.

**Table 3:** Comparison of BES scores and categories according to food addiction status.

Condition	NFA (*n* = 666 )	FA (*n* = 205)	*p* Value
	Mean ± SD	Mean ± SD	
**BES total**	8.23 ± 6.35	22.56 ± 7.93	<0.001
**BES emotions**	4.05 ± 3.71	12.76 ± 4.44	<0.001
**BES behaviours**	4.72 ± 3.66	11.56 ± 4.75	<0.001
	%	%	
**Non-bingeing**	90.1	27.3	<0.001
**Moderate bingeing**	9.0	41.0	0.046
**Severe bingeing**	0.9	31.7	<0.001
***Total***	100.0	100.0	

Note: BES, Binge Eating Survey; NFA, Non-food addicted; FA, food addicted. Differences in BES scores determined by independent samples *t*-tests, differences in proportions for BES category determined by chi squares.

**Table 4:** Pearson correlation coefficients between the BES factors and YFAS symptoms (*n* = 953).

Measure	BES Total	BES Emotions	BES Behaviours
*Binge Eating*			
1. BES total	-		
2. BES emotions	0.95 ***	-	
3. BES behaviours	0.96 ***	0.82 ***	-
*Food Addiction*			
4. Total FA symptoms	0.76 ***	0.75 ***	0.71 ***
5. Consumed more than planned	0.24 ***	0.21 ***	0.25 ***
6. Unable to cut down or stop	0.61 ***	0.62 ***	0.55 ***
7. Great deal of time spent	0.58 ***	0.55 ***	0.56 ***
8. Activities given up or reduced	0.61 ***	0.61 ***	0.56 ***
9. Continued use despite physical/emotional consequences	0.67 ***	0.67 ***	0.60 ***
10. Tolerance	0.54 ***	0.54 ***	0.50 ***
11. Withdrawal	0.52 ***	0.52 ***	0.48 ***
12. Continued use despite social consequences	0.52 ***	0.51 ***	0.48 ***
13. Fail to fulfil roles and obligations	0.53 ***	0.53 ***	0.48 ***
14. Use in physically hazardous situations	−0.49 ***	−0.50 ***	−0.44 ***
15. Craving	0.65 ***	0.64 ***	0.59 ***
16. Impairment or distress	0.68 ***	0.70 ***	0.61 ***

*** *p* < 0.001. Shading indicates correlations that are > *r* = 0.6, BES, Binge Eating Survey; YFAS, Yale Food Addiction Scale.
